# Genetic Factors Involved in Fumonisin Accumulation in Maize Kernels and Their Implications in Maize Agronomic Management and Breeding

**DOI:** 10.3390/toxins7083267

**Published:** 2015-08-20

**Authors:** Rogelio Santiago, Ana Cao, Ana Butrón

**Affiliations:** 1 Facultad de Biología, Dpt Biología Vegetal y Ciencias del Suelo, Universidad de Vigo, As Lagoas Marcosende, Vigo 36310, Spain; 2 Agrobiología Ambiental, Calidad de Suelos y Plantas (UVIGO), Unidad Asociada a la Misión Biológica de Galicia (CSIC), Pontevedra 36143, Spain; 3 Misión Biológica de Galicia (CSIC), Box 28, Pontevedra 36080, Spain; E-Mails: anacao@mbg.csic.es (A.C.); abutron@mbg.csic.es (A.B.)

**Keywords:** maize, *Fusarium verticillioides*, Fusarium ear rot, fumonisin, maize breeding

## Abstract

Contamination of maize with fumonisins depends on the environmental conditions; the maize resistance to contamination and the interaction between both factors. Although the effect of environmental factors is a determinant for establishing the risk of kernel contamination in a region, there is sufficient genetic variability among maize to develop resistance to fumonisin contamination and to breed varieties with contamination at safe levels. In addition, ascertaining which environmental factors are the most important in a region will allow the implementation of risk monitoring programs and suitable cultural practices to reduce the impact of such environmental variables. The current paper reviews all works done to address the influence of environmental variables on fumonisin accumulation, the genetics of maize resistance to fumonisin accumulation, and the search for the biochemical and/or structural mechanisms of the maize plant that could be involved in resistance to fumonisin contamination. We also explore the outcomes of breeding programs and risk monitoring of undertaken projects.

Fumonisin are among the most prevalent mycotoxins worldwide found in maize and maize based foods and feeds. *Fusarium verticillioides* and *F. proliferatum* are the most relevant fungal species for fumonisin contamination because of their ample geographic distribution, prevalence, and toxigenic capacity. Other fungal species have also been found to produce fumonisins *in vitro*, such as *Alternaria alternata* f. sp. *lycopersici* and *Aspergillus niger* [1,2]. High levels of naturally occurring fumonisins in kernels have been reported worldwide; in regions from Europe [3,4], Unites States [5,6,7], Brazil and Argentina [8,9], China [10,11], Iran [12], Nigeria and Benin [13,14] and South Africa [15].

It is known that fumonisin consumption can cause several disorders in humans and animals—leukoencephalomalacia in horses and pulmonary edema in swine, both accompanied by liver and heart damage, hepatic necrosis and kidney and liver cancer in rodents—and can impair growth in poultry and liver function in cattle, among other damages [15,16,17]. Regarding their toxic effects on humans, several epidemiological studies have related the consumption of fumonisin-contaminated maize to the high incidence of esophageal cancer in populations from South Africa, China and Iran [10,12,18], and to the occurrence of neural tube defects in human embryos in the USA after observing this effect in mice [19,20]. Fumonisins are classified as possibly carcinogenic to humans by the International Agency for Research on Cancer [21], and regulations and guidance for fumonisin concentrations in foods and feeds have been established in several countries including Europe, USA, and Brazil.

Contamination of maize with fumonisins depends on environmental conditions, maize resistance to contamination and the interaction between both factors. As natural infection of maize plants by *Fusarium* and fumonisin accumulation occur in the field, the environmental conditions during the cultivation period are decisive for the fumonisin contamination levels reached in the kernels at harvest, but plant characteristics are also relevant for both infection and fumonisin accumulation. The appropriate use of the maize genetic variability for fumonisin resistance and the avoidance of critical environmental conditions by means of suitable agronomic practices are necessary tools to reduce the contamination of kernels with fumonisin below safety levels. In this review, we address the most critical factors for the occurrence of fumonisin accumulation in maize kernels in the field, the progress achieved by plant breeding and the current knowledge on the relationship between biochemical and physical characteristics of maize plant and resistance to fumonisin contamination.

## 1. Environmental Factors Affecting Fumonisin Contamination in Maize Kernels

### 1.1. Effect of Temperature and Water Activity in Vitro Studies

As establishing the importance of each environmental factor for fumonisin contamination is very complex under field conditions due to the high number of variables changing from one location to another or from one year to another, diverse studies on the influence of temperature and water availability—the main environmental factors affecting fumonisin production—on *F. verticillioides* performance have been conducted under laboratory conditions.

In general, fumonisin production by *F. verticillioides* increases with increasing water activity (*a*_W_) in the substrate, from 0.92 up to 0.98–1.00 *a*_W_. Optimal temperature for production ranges between 20 and 30 °C, depending on the isolate, but can occur at 10–37 °C [22,23,24,25]. At optimal *a*_W_, cyclical temperature conditions, resembling those that occur naturally in the field, can favor fungal growth and fumonisin production more than isothermal conditions; thus, under daily variations between 10 or 15 °C and 25 °C, *F. verticillioides* produce more fumonisins *in vitro* than at a constant temperature of 25 °C [26,27].

Some studies reported that under suboptimal temperatures for growth (15 °C), relative fumonisin production can be stimulated at moderate *a*_W_ (≤0.95 *a*_W_) [23,27]. Similarly, moderate water stress conditions and moderate temperatures (15–20 °C) have shown to increase *F. verticillioides FUM1* gene expression [28,29,30]. Other genes of the fumonisin biosynthetic cluster, instead, are regulated differently by temperature and *a*_W_ [25]; for example, the optimal temperature for *FUM21* expression has been reported both at 15 and at 25 °C [30,31]. Field experiments also suggest that exposure to suboptimal temperatures and water availabilities that can occur during kernel drying may trigger fumonisin biosynthesis [32].

### 1.2. Environmental Factors Affecting Fumonisin Contamination in the Field

#### 1.2.1. Temperature, Air Humidity and Rainfall

It is considered that, in temperate zones, *F. verticillioides* and fumonisin contaminations are predominant in maize kernels in warmer and drier regions, and conversely, other mycotoxigenic *Fusarium* species such as *F. graminearum* or *F. subglutinans* develop better in cooler or/and wetter climates [3,32,33,34,35,36,37,38,39,40,41]. The fumonisin producer *F. proliferatum* is less distributed and often co-occurs with *F. verticillioides* in maize ears in warmer regions in lower frequencies, although relevant levels of *F. proliferatum* have been reported in some regions of southern Europe, central Argentina, Benin or Iran [8,14,42,43,44,45]. However, there is not a direct relationship between dryness and temperature and kernel contamination with fumonisins across different environments. For example, in temperate Spain, contamination of maize kernels with fumonisins is generally very low in the dry and warm areas of the northeast (≤1 µg/g) [46,47], while, in the mild and wetter areas of the northwest, fumonisin levels are higher and closer to the safety levels of 4 µg/g established by the European Union for human consumption [4,32]. In surveys located in tropical Africa, *F. verticillioides* and fumonisin abundances have been related to warm and less wet regions in Zambia [48], but to more humid regions in Benin, Zimbabwe, or Uganda [14,49,50]. Fandohan *et al.* [14] suggested that fumonisin contamination in the more humid regions of Benin may be favored by more than one crop per year, considerable insect infestation and fungal infection in the field. Similarly, in Uganda, the higher fumonisin contamination in maize from high altitude fields was attributed to higher humidity and inadequate agronomic practices [50].

In addition to geographical variation for fumonisin contamination, occurrence of *F. verticillioides* and fumonisins in maize kernels can vary greatly from one year to another. Although greater fumonisin contaminations were often associated to warmer or drier years [5,6,38,48,51,52], some studies have pointed out the lack of a clear association between drought and fumonisin contamination because years with combined conditions of warm weather and high precipitation before harvest resulted in higher fumonisin accumulation, or greater fumonisin levels occurred associated to higher humidity or rainfall independently of temperature conditions [53,54,55,56,57,58]. For example, in a three-year evaluation in northern Italy conducted by Maiorano *et al.* [59], lower fumonisin contamination was found in the year with the driest and warmest conditions during the flowering and ripening months while higher contamination occurred in the year with higher rainfall in the same periods.

General climatic characteristics cannot satisfactorily explain differences for fumonisin contamination among environments, because the fungus develops inside maize kernels which suffer dramatic changes during the processes of kernel filling and drying. Moreover, the impact of external variables on those processes, as well as their direct impact on the fungus, will determine the amount of fumonisin in maize kernels at harvest. Meteorological patterns throughout the season, insect and fungal diversity, or cultural practices have been identified as determinants for the final amount of fumonisin in maize kernels.

#### 1.2.2. Critical Periods during Maize Development

Climatic characteristics during two periods of maize development, flowering and kernel drying, seem to be critical for kernel contamination with fumonisin [60,61,62,63,64]. In general, low rainfall and hot temperature around flowering, and high rainfall or high temperature just before harvest, were found conducive for fumonisin contamination. The exposed silks are the main pathway for *F. verticillioides* to naturally enter into the ear and reach the kernels, although kernels wounds made by insects or other biotic or abiotic agents could also favor kernel infection [7,37,60]. In a wide survey across different locations in USA, fumonisin contents were inversely correlated with precipitation near flowering [61]. A critical period around silking was also identified by de la Campa *et al.* [62] in a study developed in Argentina and Philippines. Fewer days of precipitation >2 mm from two to 14 days after silking resulted in higher contamination levels and explained most of the variability in fumonisin content, followed by more days of maximum temperatures >34 °C and fewer cool days of minimum temperatures <15 °C around silking. Similarly, in an evaluation conducted in northwestern Spain, maximum temperature around the flowering period was the most influential variable; specifically, more days of maximum temperatures ≥30 °C increased fumonisin contamination [63]. Precipitation, humidity, and cool days around flowering, however, were negatively related to *F. verticillioides* infection and fungal growth, but not to fumonisin contamination [63]. In accordance with these results, Maiorano *et al.* [64] reported that rain during flowering can be favorable for fumonisin contamination when daily rain intensity is below 2 mm/h. Rain and humidity seem favorable conditions to kernel infection by *F. verticillioides* and the consequent contamination with fumonisinsm, since water splashing disperses fungal propagules and moisture favors spore germination and mycelial growth; however, hard rain could limit spore dispersal and wash off inoculum reservoirs counteracting the positive effect of wetness [22,65].

Some authors have pointed out that drought stress in maize during flowering increases susceptibility to fungal infection and insect attack [51,66]. However, under controlled irrigation, drought stress did not seem to be an important factor for fumonisin accumulation in maize kernels [7,57,67,68]. It has been alternatively suggested that dry and warm conditions during flowering could favor the movement of insects inside the ears, and, consequently increase the chance of fumonisin contamination [7,37].

The other critical period for fumonisin accumulation is the kernel drying period. Kernel colonization by *F. verticillioides* can start a few weeks after flowering and trace amounts of fumonisins can be detected in kernels from the blister-milk stage [32]. The dent stage appears to be the most conducive for fumonisin biosynthesis [69] and establishes the starting point for fumonisin accumulation, which reaches the highest amounts after physiological maturity during kernel drying in the field [8,32,52,69]. Precipitation during the weeks before harvest has been associated with greater *F. verticillioides* incidence and subsequent fumonisin contamination [37]. In this regard, more moderate-hard rainfall during the kernel drying period has been shown to contribute to increased fumonisin contamination at harvest [63]. Rainfall probably maintains moisture conditions conducive for fumonisin production inside the kernels because kernel drying is slowed down. Other reports, instead, found no positive correlation between rainfall before harvest and fumonisin levels [48], or that maximum temperature in the months preceding harvest was a more influential variable than precipitation comparing different localities [41].

#### 1.2.3. Fungal Diversity

It seems obvious that the primary factor for fumonisin contamination is the presence of fumonisin-producing fungi. Most *F. verticillioides* strains are able to produce fumonisin, and there is a wide variability in their production capacity and pathogenicity [18,70,71]. Under natural inoculation conditions, symptomatic kernels usually have the highest contents of fumonisin, but relevant contents can be obtained from kernel samples with low levels of visible infection [52,70]. This can be related to the endophytic behavior of *F. verticillioides* and the fact that fumonisins are products of the secondary metabolism, so fumonisin production and fungal growth can respond differently to environmental conditions [22]. In addition, the interaction of *F. verticillioides* with other fungal species in maize kernels may affect *F. verticillioides* growth and fumonisin production. *In vitro*, some *Aspergillus*, *Penicillium*, *Fusarium*, *Alternaria* and *Thrichoderma* species have been found to inhibit or enhance fumonisin production by *F. verticillioides* [22]. In field studies, the co-occurrence of *F. graminearum* and *F. verticillioides* in the ears did not change the net content of fumonisin in the kernels [69,72]. Studies conducted with *A. flavus* showed some competition effects on *F. verticillioides* growth, although aflatoxin and fumonisin levels were found positively correlated in maize ears naturally infected [6,73,74].

#### 1.2.4. Insect Infestation

Frequently, visible mycelium grows around and from kernel wounds produced by insects. Many insect species have been associated with fungal diseases since their activity disperses the fungus and provides routes of entry into the ear and kernels [75,76,77]. In addition, some common pest species of maize have been directly related with fumonisin contamination such as the European corn borer (ECB; *Ostrinia nubilalis*) [58,67,78,79] and the Mediterranean corn borer (MCB; *Sesamia nonagrioides*) [80]. Other species associated with fumonisin contaminations and responsible for kernel injuries are corn earworm (*Helicoverpa zea*) and ear thrips (*Frankliniella spp*.), which showed low and high correlations, respectively, with fumonisin content in kernels [7,81,82], Angoumois grain moth (*Sitotroga cerealella*) [32,63], fall armyworm *(Spodoptera frugiperda*) and sap beetles (*Carpophilus spp*.), among others [83]. Damages caused by *Diatrea saccharalis*, *H. zea* and *O. furcanalis* contributed moderately to the variability for fumonisin contents in maize kernels in the study conducted by de la Campa *et al.* [62], and natural ear damage by MCB was the most influential environmental factor affecting fumonisin contamination in evaluations in northwestern Spain [63]. The use of Bt hybrids and insecticide treatments achieved significant reductions in the concentration of fumonisin in kernels supporting the hypothesis that insect injuries have a relevant effect on contamination [78,79]. The use of maize genotypes resistant to ear damage could contribute to the reduction of fumonisin contamination in the kernels, however, in particular environments, other factors can influence fumonisin contamination more than damage by borers [84].

#### 1.2.5. Agronomic Practices

The application of suitable agronomic practices is a tool for modulating the effect of environmental conditions conducive for fungal infection and fumonisin accumulation and minimizing the risk of contamination [85,86]. An appropriate choice of sowing and harvest dates may help in avoiding adverse conditions during the critical periods, flowering and kernel drying. In temperate zones, earlier sowings often result in lower fumonisin contamination of maize kernels than later sowings [53,64,79,87]. Earlier sowings reduce the risk of overlapping the flowering with drier and warmer weather, when the environmental conditions are the most favorable for fungal spread and ear colonization by some insect species such as thrips and corn earworms [7], and bring forward the ear development avoiding the period with more ECB activity [79]. On the contrary, in environments where Angoumois grain moth incidence is an important factor, maize sown earlier has shown to be more susceptible to kernel damage and subsequent fungal and fumonisin contamination, possibly due to it reaching sooner the threshold kernel moisture that allows moth infestation [32,63].

On the other hand, maize harvested earlier usually presents lower fumonisin contamination levels. Early harvests reduce the time that fumonisins are accumulated in the field and exposure to seasonal rainfalls that can delay kernel drying [32,52,57,59,63,87].

Insecticide treatments, removal of debris, or moderate nitrogen fertilization are other proposed practices to reduce fungal infection and fumonisin accumulation [59,79,86,88]. Plant density, irrigation regimes and tillage showed no consistent effects on fumonisin contamination [7,57,59,68,88], and fungicide treatments are not effective in reducing fumonisin in kernels, except when combined with an insecticide treatment [44,58]. Contrary to what has been observed in other cereals, maize cultivated in organic agriculture does not accumulate less fumonisins than conventional maize [46,89].

### 1.3. Modeling Fumonisin Contamination

The use of models to predict fumonisin accumulation in maize becomes an integrative tool which incorporates different genetic and environmental factors required to assess the risk of exceeding safety levels. Several predictive models have been proposed for *F. verticillioides* infection and fumonisin contamination by including different combinations of climatic, agronomic and maize genotype factors.

De la Campa *et al.* [62] developed a multiple regression model for predicting fumonisin contamination with two year data from Argentina and Philippines. They focus on the weeks around silking and identified four periods between 10 days and 14 days after silking where weather variables were critical. The weather variables used were days with maximum temperatures >34 °C, minimum temperatures <15 °C and rainfall >2 mm, and were introduced as binary values. When the insect damage was included, the model explained 82% of the variation for fumonisin amounts. Analyzing several temporal windows with silking as reference, Martínez *et al.* [90] developed a logistic regression model with data from Argentina including only meteorological variables and their interactions: daily maximum and minimum temperatures, rainfall and relative humidity. The most critical period was set between seven days and 10 days after silking, and the most influential variables, positively related to fumonisin, were based on a combination of rain and relative humidity. From a different approach, Battilani *et al.* [87] constructed a logistic regression model by considering only cultural practices, with data collected over six years from fields in northern Italy. The crop system parameters recorded were soil texture, previous crop, debris management, tillage and other field operations, hybrid seeded, sowing period and investment, mineral nutrition, weeds control, irrigations, flowering period, crop injuries (borers, hail, and wind), chemical control of ECB, harvesting period, and moisture of kernels at harvesting. Planting maize hybrids with a maturity class below 128 days and harvesting at or before the 24^th^ week after planting greatly reduced the risk of fumonisin contamination.

Torelli *et al.* [57] tried a neural network model to predict fumonisin contamination based on several agricultural parameters in a more restricted region. Data were obtained from two year evaluations in northeast Italy and parameters included irrigation regime, ECB treatment, sowing and harvest dates, water content at harvest, crop duration, FAO class, years and locations. The model obtained achieved positive and moderate correlations between observed and predicted data.

Maiorano *et al.* [64] developed a comprehensive risk assessment model of fumonisin production in maize kernels on the basis of the maize-*F. verticillioides*-ECB pathosystem, constructed with bibliographic and experimental data collected in northern Italy over four years. Meteorological data related to temperature, relative humidity, wind speed, and rain intensity were used to construct the model as influential factors for maize plant phenology, fungal development, and insect activity. Sowing dates, flowering, *a*_W_ of silks and kernels, and dry-down information for each maize hybrid cultivated, and the application or not of chemical treatments against ECB were also included. Parameters affecting infection during flowering had the greatest weight on fumonisin contamination, followed by the parameters affecting ECB damage, and the parameters related to fumonisin biosynthesis in kernels.

More recently, Cao *et al.* [63] assessed fumonisin contamination and *F. verticillioides* infection and growth on the basis of a factorial regression model involving environmental and genotype factors in a three year study in northwestern Spain. A wide set of climatic variables were measured in several temporal windows along the entire growing period, including the critical periods around flowering reported by de la Campa *et al.* [62], insect pressure and kernel damages. Plant characteristics, such as husk tightness, pericarp thickens or resistances to insect injuries were included as genotypic factors. Most of the variation for fumonisin contamination was explained by environmental factors; fumonisins were positively related mostly to ear damages by borers (mainly MCB), followed by days with maximum temperatures ≥30 °C during the flowering period (between 15 days before and 15 days after flowering), and days with daily rainfall ≥2 mm during the drying period. Genetic characteristics of the hybrids tested had a smaller influence on fumonisin accumulation than environmental factors; but maize resistance to damage by the Angoumois grain moth and thinner pericarps reduced fumonisin accumulation.

However, maize genetic variability for fumonisin contamination and the probability of success in achieving higher resistance by implementing breeding programs have to be assessed in field experiments under artificial inoculation in order to guarantee a homogeneous distribution of the pathogen.

## 2. Maize Breeding for Resistance to Kernel Contamination with Fumonisins

Plant breeding has emerged as an effective and environmentally safe method to control fungal infection and reduce mycotoxin levels in susceptible crops [86,91]. Higher resistance levels and reduced fumonisin contamination in maize kernels are highly possible considering the high genetic variability observed for resistance to fumonisin accumulation and its moderate to high heritability [92,93,94,95,96]. However, direct selection for reduction of mycotoxin contamination is expensive and time consuming, so indirect, cheaper and less time consuming selection criteria are necessary [97]. In this context, Fusarium ear rot has emerged as a suitable trait for performing indirect selection because high genotypic correlations between Fusarium ear rot and fumonisin contents have been reported [92,95,96,97,98].We also include those works focused on exploring genetic variability for Fusarium ear rot and/or to study maize genetics involved in resistance to Fusarium ear rot.

### 2.1. Sources of Resistance to Fusarium Ear Rot and Fumonisin Contamination

The search for sources of resistance can be done under natural inoculation when dispersion and quantity of inoculum is guaranteed, but the identified sources of resistance should be checked again under artificial inoculation. For example, two hybrids were identified as resistant to fumonisin contamination out of 10 hybrids evaluated under natural inoculation [4], but only the inbred EP10 × EC22 confirmed its resistance under artificial inoculations with *F. verticillioides* [27]. A detailed review on inoculation issues such as the type and quantity of inoculum, the method of inoculation and the best time for performing inoculations with *F. verticillioides* can be found in Mesterházy *et al.* [99]. The ideal inoculation technique must result in a sufficient level of infection to differentiate among genotypes for resistance but to be below the infection threshold at which differences become difficult to observe. The inoculation techniques most often tested include inserting a *Fusarium*-colonized toothpick into the ear or the silk channel, pinbar inoculation, spraying a spore suspension onto silks, and injecting a spore suspension down the silk channel or through the ear husks into the kernels [99,100,101,102]. Relevant authors have concluded that techniques involving injections in kernels or the silk channel are the most efficient to distinguish among genotypes resistant to Fusarium ear rot and fumonisin accumulation and appeared to be the best alternatives for artificial inoculation with *F. verticillioides* [91,100,102]. The study by Schaafsma *et al.* [103] suggested that the kernel inoculation method would be more suitable when the goal is to screen genotypes for their resistance to fumonisin accumulation by using Fusarium ear rot as an indirect selection criterion, because consistent correlations between these two traits were only found after kernel-wound inoculation. However, other studies showed high and significant genetic correlations, ranging from 0.76–1.00, between fumonisin content and Fusarium ear rot severity under silk channel inoculation [93,96]. A wide maize genetic diversity was evaluated under artificial inoculation with *F. verticillioides* in experiments located in Africa, Europe and America and sources of resistance to Fusarium ear rot and fumonisin contamination have been found among hybrids [54,100,104,105,106,107], inbreds [13,94,95,96,106,108,109,110,111], and landraces [112].

### 2.2. Inheritance of Maize Resistance to Fusarium Ear Rot and Fumonisin Contamination

Genetic architecture of resistance to ear rot by *F. verticillioides* and fumonisin contamination appears complex with many quantitative trait loci (QTL) of small effects controlling each trait; some of them with possible pleiotropic effects on both resistance traits [113,114,115,116,117,118,119]. In addition, meta-QTL analyses have shown that genes for improving resistance to kernel infection by *F. verticillioides* and/or fumonisin accumulation could have pleiotropic effects on resistance to other mycotoxigenic fungi such as *Aspergillus flavus* and *F. graminearum* or to be linked to genes for resistance to those species. On this sense, although breeding for resistance to fumonisin accumulation would be hampered by the quantitative inheritance of the trait, improved varieties could have effects on resistance to kernel accumulation by other mycotoxins [113,117]. Xiang *et al.* [113] reported two meta-QTL for Gibberella and Fusarium ear rots in chromosome 1, three in chromosome 2, four in chromosome 3, one in chromosome 4, two in chromosome 5, one in chromosome 6, and two in chromosome 7; they also reported 9 meta-QTL for Aspergillus and Fusarium ear rots in chromosomes 3, 4, 5, and 6. In a further work, Mideros *et al.* [117] localized 10 meta-QTL for resistance to fumonisin and aflatoxin accumulation on chromosomes 1, 3, 4, 5, and 8. In recent genome-wide association studies, several SNPs were significantly associated to Fusarium ear rot, but the value of selection of these SNPs for improving resistance to Fusarium ear rot is limited because each SNP explained only small percentages of trait variation [120,121].

Simultaneously, candidate genes for maize resistance to Fusarium ear rot have been proposed in transcriptome studies deployed to study maize response to infection to *Fusarium verticillioides* [122,123,124,125,126,127]. In these studies, maize genes specifically involved in maize response to *F. verticillioides* infection have been identified in resistant and susceptible inbreds. These genes can be considered as valuable resources to undercover maize resistance mechanisms to Fusarium ear rot. Moreover, resistance of some genotypes may be mainly due to constitutive defense mechanisms such as the constitutive reinforcement of cell walls by lignin and/or other phenolic compounds.

QTL [115,116,119], inbred diallel [128,129,130,131], inbred testcross [111] and generation-mean [131,132] studies have addressed the importance of each genetic (additive, dominance and/or epistasis) effect on maize resistance to Fusarium ear rot and fumonisin accumulation; additive effects have been reported as the most important for the inheritance of resistance to Fusarium ear rot and fumonisin accumulation, but dominance as well as epistatic effects could play an important role in the inheritance of these traits [111,115,116,119,128,129,130,131,132,133,134]. Results from an 18-inbred line (inbreds belonging to different heterotic groups and showing different levels of resistance) diallel suggested that the most efficient way to improve Fusarium ear rot and fumonisin contamination would be to evaluate and select among inbreds before using resources to create and evaluate hybrids because genetic variation among inbreds was higher than among hybrids and a high and significant correlation coefficient (*r* ≥ 0.78) was found between *per se* performance of inbred lines and their general combining abilities (GCA) [129]. However, other authors [92] recommend doing selection for testcross performance of inbreds due to moderate genotypic correlation coefficients between lines and testcrosses.

Although significant variances for the genotype-environment interaction have been reported for Fusarium ear rot and fumonisin content [92,135], genotypes tended to show stability for both traits across different environments even when genotype evaluations were done in a wide range of environments [93,129,136]. Robertson *et al.* [93] attributed the genotype-environment significant effects to heterogeneity of genotypic variance, rather than to the lack of correlation of genotype performance in different environments. Similarly, Butrón *et al.* [132] stated that genetic effect-environment interactions for Fusarium ear rot and fumonisin contamination could be attributed to differences of magnitude of the main genetic effects (additive and dominance) among environments, although the involvement of some specific genomic regions (QTL) in maize resistance could depend on the environment [114,115,116].

### 2.3. Breeding Programs for Reducing Kernel Contamination with Fumonisins

In the USA, concerns of industry inspired researchers of the University of Illinois to initiate a breeding program for kernel resistance to fumonisin accumulation and Fusarium ear rot in the late 1990s [137]. In 1999, they developed over 1500 F_1_ hybrids using a large, genetically diverse collection of inbred lines that were crossed to the elite inbred FR1064 in order to find valuable sources of resistance. In later years, they developed F_2_, and BC_1_ generations from all F_1_ hybrids and F_3_ and BC_1_S_1_ generation from selected genotypes for performing studies of inheritance of resistance and for identifying molecular markers associated with resistance. To complement the search for sources of resistance performed by the University of Illinois, researchers at the North Carolina State University began to do screening trials for both Fusarium ear rot and fumonisin concentration. They used materials containing tropical germplasm selected by Dr. Mike Blanco from the Germplasm Enhancement of Maize (GEM) project or developed by Dr. Major Goodman’s breeding program at North Carolina State University [91], and reported the effectiveness of a backcrossing program for improving quantitatively inherited disease resistance traits based on phenotypic evaluations [97]. The North Carolina State University team also performed QTL studies that concluded that marker-assisted selection cannot be generally recommended for these traits, but marker-assisted backcrossing may be efficient to transfer alleles from resistant but agronomically poor lines to elite inbreds [91,116], although more recent studies, based on genome wide association, have suggested that marker-assisted introgression of resistance alleles from unadapted subpopulations should be done in combination with genomic selection for the polygenic background for both the target trait and general adaptation traits [120,121].

In many parts of the world, public pedigree selection programs have also been successful in improving resistance to ear rot caused by several fungi, including *F. verticillioides,* either doing intentional or unintentional phenotypic selection [130,138,139,140]. In parallel, the private industry began to release inbreds and hybrids with above average resistance to Fusarium ear rot according to the seed patents from Pioneer Hi-bred Int Inc approved since 1997 [141,142,143,144,145,146,147]. More interestingly, breeding for resistance to one mycotoxigenic species could affect resistance to other ones [98,130,132].

## 3. Genotypic Traits Influencing Fusarium Ear Rot and Fumonisin Contamination

The best way to reduce or prevent fumonisin contamination is to limit their biosynthesis during cultivation of maize plants. Identifying maize factors associated with resistance to infection by *Fusarium* and fumonisin accumulation helps in the understanding of genetic mechanisms controlling resistance and also facilitates maize breeding because indirect selection on characteristics strongly associated to resistance can be performed. Maize cultivar characteristics, such us precocity, husk coverage, silks duration, or the chemical composition of the kernels may influence fungal infection and subsequent fumonisin production.

### 3.1. Precocity of the Plant

Attending to maturity, disease may be minimized on ears of early-maturing inbreds and hybrids if kernels mature quickly and kernel moisture drops rapidly below levels that are favorable for growth and sporulation of *Fusarium* spp. [148,149]. Late-maturing maize cultivars in which grain moisture content decreases slowly below 30% are most susceptible to *Fusarium* disease [150]. Hybrid maturity class was the cultural factor with the greatest effect on fumonisin contamination in a six-year study in northern Italy performed by Battilani *et al.* [87]. However, some other studies noted that the final fumonisin concentration in maize kernels does not seem to be primarily influenced by hybrid maturity [55,151,152,153,154]. In this regard, Battilani *et al.* [154] pointed out that hybrids with the slowest kernel drying rate were the most favorable for fumonisin accumulation, irrespective of their maturity classification. The variation of water content in maize kernel during ripening (measured as *a*_W_ and kernel moisture) influences fumonisin production and can be used to predict contamination, but is not related to the FAO class of the hybrids tested. Factors, such as air temperature and hybrid characteristics such as thickness of the kernel pericarp_,_ may be more decisive for the water loss rate and, hence, for fumonisin accumulation in kernels than hybrid maturity classification [64,154].

### 3.2. Husk Coverage

The presence of fumonisins seems to be strongly linked to maize characteristics, among them, the husk coverage has been consistently implicated in the susceptibility of maize hybrids to infection by *F. verticillioides*. Failure of the husk to protect the tip permits easier entry of insects and consequent fungal contamination [77,155,156,157,158,159,160]. Duncan and Howard [161] showed that growth and sporulation of *F. verticillioides* was evident only on the exposed pollinated silks, with a significant growth reduction on silks that were covered by the husk. Up-to-date, Butrón *et al.* [4] noted how hybrids with tighter husks and less exposed ear tips present more resistance to *Fusarium* infection.

### 3.3. Silks’ Characteristics

As the maize silks are considered to be major routes for entry of *Fusarium* species into non-damaged ears [155,162], physical and/or chemical characteristics of the silks and the silk channel could be key factors of resistance against the fungus. Traits such as delayed silk senescence could contribute to resistance of some genotypes by imposing a physical barrier between kernels and inoculum sources [149,163,164]. Brown silks may have been limited in moisture, resulting in slowed fungal growth, a lack of extensive surface colonization, and inhibition of development of symptoms on kernels. Several studies [165,166,167] reported field results of reduced fungal ear rot correlated with accelerated silk senescence and longer intervals of time between silk emergence and silk channel inoculations. Lately, a strong correlation has been reported between levels of fumonisin contamination in kernels and duration of silking and silk wetness [64].

Plant defenses consist of physical barriers as well as chemical defense mechanisms that are induced in response to external stimuli. Results from Sekhon *et al.* [168] agree with those of Reid *et al.* [169] and suggest that the amount of phenolic and flavones present in the non-inoculated silk tissue is not a good indicator of maize resistance to Fusarium ear rots. However, upon fungal inoculation, some induction of 3-deoxyanthocyanidins was observed in the resistant line suggesting the role of these compounds in resistance to *F. verticillioides* [168].

### 3.4. Kernel characteristics

#### 3.4.1. Color

In the *p1* locus the dominant *P1* allele provides pigmentation selectively to plant floral organs, in particular pericarp and cob, due to accumulation of phlobaphene pigments derived by polymerization of flavan-4-ols [170]. Accordingly, Pilu *et al.* [171] indicate that the accumulation of flavonoid pigments in the seeds, in particular phlobaphenes, is able to reduce the level of fumonisins. Those authors conjecture that flavonoid compounds produced in high quantities by the presence of regulatory factors such as *R1*, *B1*, *Pl* and in particular *P1* could act in some way to lower the fumonisin B1 accumulation. In these sense, the pigment accumulation is linked to a reduction of corn borer attacks and/or fungal infections and consequently mycotoxin accumulation.

Shephard *et al.* [172] reported that in some years, fumonisin levels were significantly lower in yellow than in white maize, but the reverse situation was observed in other years; on the other side, Santiago *et al.* [94] noted that genotypes with white kernels presented higher fumonisin concentration at the 0.10 probability level. However, although white maize inbreds had higher levels of fumonisin than yellow maize inbreds, it was still possible to find white inbreds with comparable resistance to fumonisin accumulation to that of the most resistant yellow inbreds indicating that some other resistance traits are involved.

#### 3.4.2. Antioxidant Profile

One of the first biochemical events following the plant infection by a pathogen is the production of reactive oxygen species (ROS), including hydrogen peroxide (H_2_O_2_), involved in many signaling transduction pathways. This mechanism induces perturbation of the oxidative state of the plant cell which may interfere with the fungus metabolism. Picot *et al.* [173] evaluated the potential involvement of antioxidants (α-tocopherol, lutein, zeaxanthin, β-carotene, and ferulic acid) in the resistance of maize varieties to Fusarium ear rot. On the basis of that study, α-tocopherol was to be a more potent inhibitor of fumonisin production than ferulic acid. Nevertheless, *in planta*, the latter is more abundant than the former, at any kernel stage. In this sense, ferulic acid has been previously described as a potent repressor of mycotoxins biosynthesis [174], although the *in planta* mode of action on fungal growth and/or fumonisin accumulation has yet to be elucidated.

#### 3.4.3. Lipidic Profile

Shelby *et al.* [61] tested 15 maize hybrids and found no significant correlation between lipid content and fumonisin contamination. Nevertheless, contemporary studies have shown that the lipidic composition of maize kernels can influence fumonisin content. Polyunsaturated fatty acids, released from membranes by lipases in response to attacks by biotic agents, play a key role in plant-pathogen interaction either directly as free fatty acids or as precursors of oxylipins [175]. The key role played by fatty acids in the regulation of fumonisin production in maize has been suggested [176]. Fatty acids, such as linoleic acid, and some of its oxylipin-derivatives [e.g., 9-HODE] produced by the host in response to the attack of a mycotoxigenic fungus may trigger the synthesis of fumonisins [177]. In particular, Gao *et al.* [178] reported that, in a 9-oxylipin-deficient maize mutant, fumonisin produced by *F. verticillioides* was reduced by 200-fold. 9-HODE is produced by maize ears during ripening under field conditions and it is present in a higher amount in those maize samples with high fumonisin contamination [179].

#### 3.4.4. Proteic Profile

Proteomic studies represent an alternative approach to evaluate the plant defense response to pathogen infection. In maize, few detailed molecular studies are available on its response to *F. verticillioides* infection. Induction of specific pathogenesis-related (PR) proteins and protein-kinases following fungal infection are reported [180,181]. Campo *et al.* [181] performed experiments in maize embryos to identify the response to *F. verticillioides* infection at the protein level. Different types of antioxidant enzymes were detected, such as Cu/Zn-superoxide dismutase, glutathione-S-transferase and catalase, which normally protect cells from oxidative damage. Proteins involved in the initiation of other protein synthesis or which participate in protein folding and stabilization were also identified [181]. Changes in detoxification enzymes, lipid transfer proteins, ribosomal proteins, aldolases, dehydrogenases, glucanases and chitinases have been also reported [123,125,181,182]. In addition, proteins with significant effect on aflatoxin accumulation resistance, such as glyoxalase, trypsin inhibitor, late embryogenesis abundant proteins, and heat shock proteins, could also contribute to the resistance to *F. verticillioides* [118,183].

#### 3.4.5. Pericarp (Protection Tissue)

Trying to elucidate a possible path for *F. verticillioides* entrance into the pericarp, Duncan and Howard [161] noted that the stylar canal would represent the only route to the pericarp cells from outside of the kernel in the absence of injury. They suggest that it may be useful to sort kernels of maize lines according to stylar canal architecture and then compare the groupings with kernel rot severity data observed under various conditions. However, the methodological complexity of these determinations made such assessments at a large scale unviable.

Once pathogens enter into the ear, they have to pass through the pericarp tissue to gain access to the whole kernel. Therefore, pericarp may also play an important role as a barrier against fungal invasion. In later stages of kernel development, the pericarp is composed of dead cells that are cellulosic tubes [184]. The arrangements of these thick-walled cells account for the considerable strength of the pericarp. A thin pericarp might give the fungus greater access to the kernel, especially through minor wounds caused by feeding of insects. Hoenish and Davis [185] determined that thickness of the pericarp of kernels from intermediate and resistant groups of hybrids was significantly greater than the thickness of the pericarp of the susceptible hybrids. However, results from Ivic *et al.* [186] showed that no correlation exists between pericarp thickness and resistance in Croatian genotypes, and Cao *et al.* [63] noted that a thicker pericarp favored fumonisin accumulation. A thicker pericarp could slow down the kernel drying, moisture conditions within the kernel being longer favorable for fungal growth and fumonisin production.

Wax content in outer pericarp layers and wax composition were identified as kernel factors in maize resistance to *A. flavus* infection and aflatoxin accumulation [187]. Removing wax from the pericarp significantly increased fumonisin concentration and higher wax content on kernels was associated to lower fumonisin accumulation [188]. High wax content would be a broad base resistance mechanism in maize kernels against mycotoxin production by pathogenic fungi. However, it cannot completely explain the resistance observed, suggesting that other pericarp (phenolic composition) or inner kernel factors are also involved in resistance to fumonisins.

With that assessment in mind, a wide variety of phenolics have been implicated in several biological processes including resistance or tolerance against abiotic and biotic factors [189]. Among them, the most common hydroxycinnamates found in a wide range of grasses, cell wall ferulates (FA) and *p*-coumarates (*p*CA). Cell wall hydroxycinnamates are derived from the phenylpropanoid pathway, which originates from phenylalanine and tyrosine. The diferulates (DFAs) are formed during cell wall deposition and lignification by peroxidase mediate coupling of ferulate monomers. They crosslink cell wall polysaccharides conferring pericarp hardness [190]. Hence, high contents of pericarp DFAs might act as a preformed structural barrier restricting fungal infection and mycelial progress from diseased to pericarp-intact neighbouring kernels. On the basis of the stepwise linear regression model, variability of fumonisin accumulation was best explained by 8-5’-DFA benzofuran, total DFAs and *p*CA [191]. On average, contents of *p*CA and FA were four- and two-fold higher in moderately resistant genotypes than in the susceptible ones, and were negatively correlated to fumonisin accumulation and disease severity [191]. Cell wall DFAs also might have a direct inhibitory effect on mycotoxin production after ferulic acid released by fungal esterases and other extracellular enzymes during infection of *F. verticillioides*. The 8-5′-DFA benzofuran, the major DFA detected in the pericarp of the genotypes, showed *in vitro* to be as effective as ferulic acid to inhibit the biosynthesis of trichothecenes by *F. graminearum* [174].

#### 3.4.6. Endosperm (Storage Tissue)

Hard hybrids could be connected both to a more compact pericarp which is a more effective physical barrier to fungal infection and to a greater hardness and density of the endosperm fraction that could be a less susceptible substrate to toxinogenesis [192]. In this sense, grain hardness significant influenced fumonisin content with hard endosperm hybrids showing 50% lower contamination than soft hybrids [153].

*Zea mays* var. *indentata* (dent) and *Z. mays* var. *indurate* (flint) are among the most common types of maize cultivated in Europe [193]. They differ in various properties such as plant growth, ear number per stem, ear size, and vegetation period, but particularly in morphology and anatomy of kernels. Kernels of flint type are characterized by a hard outer endosperm layer enclosing the soft endosperm, while dent maize does not contain the mentioned layer at the kernel top [194]. Flint maize showed higher resistance to fungal infection and fumonisin contamination than dent maize [94,104,151,195,196] which was attributed to higher amylase contents in kernels of the former one [197]. However, the opposite situation was also reported [135], whereas Hennigen [198] did not note any significant differences in the degree of contamination between genotypes of flint and dent maize.

Concerning the chemical composition, one of the most striking changes during kernel development is the rapid accumulation of starch when kernels mature. The starch-rich endosperm of mature kernels supports significantly higher levels of fumonisin production by *F. verticillioides* than the protein- and lipid-rich tissues of the embryo [199]. In this sense, numerous mutants with abnormal patterns of starch and proteins have been identified: maize mutant *waxy* (*wx*), *amylose extender* (*ae*), *opaque*-2 (*o2*), *sugary* 1 (*su1*), *sugary enhancer* 1 (*se1*) and *shrunken* 2 (*sh2*). Native maize starch from normal hybrids consists of amylose (27%) and amylopectin (73%) which each have a unique set of physical properties and macromolecular organization [200,201]. Mutant *wx1* kernels are characterized by the accumulation of 100% amylopectin [202,203,204], while kernels of the *ae1* mutant accumulate more than 50% amylose [204]. *Waxy* maize is more prone to fumonisin accumulation compared to the counterpart hybrids [94,205]. Diverse reasons can be pointed out: (i) the *waxy* maize starch granule sizes are slightly higher than those found in normal maize with a less compact and more branched structure [206], that should influence the penetration and colonization of the fungi into the core of the kernel; (ii) less hardness and density of the most external endosperm fraction can be a substrate more susceptible to toxinogenesis [207,208]; (iii) the lower dry down of *waxy* maize could maintain more favorable conditions for fungal colonization and growth [209]; and (iv) the presence of elevated content of amylopectin induces fumonisin production by *F. verticillioides* during colonization of maize kernels, since it affects the expression of fungal genes involved in fumonisin biosynthesis [210]. However, it must be noted that in field experiments maize kernel development was characterized by a rapid accumulation of amylopectin during the first stages of kernel development, while the enhancement of fumonisin production appeared three weeks after completion of the kernel with amylopectin, suggesting that amylopectin is not a sufficient condition to favor fumonisin biosynthesis but that other mechanisms may be involved [69].

The presence of the *opaque-2* gene (*o2*), which increases the lysine and tryptophan contents in the endosperm, make the plant more susceptible to *F. verticillioides* [211,212]. However, Santiago *et al.* [94] noted that *opaque* inbreds have significantly less fumonisin accumulation than their corresponding wild versions.

Harboring the *su1* and *se1* mutations accumulates high levels of glucose in kernels during milk and dough stages of development; however, kernels contain starch as a carbohydrate reserve at maturity [213]. Mutant *sh2* encodes ADP-glucopyrophosphorylase, which mediates the rate-limiting step in starch formation; disruption of *sh2* leads to the accumulation of glucose rather than starch in mature kernels [214]. It has been suggested that the high sucrose content in kernels of *sh2* inbreds increased infection by *F. verticillioides* [215,216]. However, studies by Headrick *et al.* [149] determine that none of the individual carbohydrate tested was related to the *F. verticillioides* infection.

In summary, our current knowledge shows that the contamination of maize with fumonisins is generally a consequence of complex interactions among diverse environmental factors, including climatic conditions (such as temperature, air humidity and rainfall), insect infestation and agronomic practices, and maize cultivar characteristics, such as precocity, husk coverage, silks duration, or the chemical composition of the kernels. The genetic architecture of resistance to fumonisin contamination appears complex with many quantitative trait loci of small effects controlling each trait. However, maize breeding that is successful in achieving higher resistance to fumonisin accumulation will be possible considering the high genetic variability observed for this species.
